# Circulating miR-134 in mesial temporal lobe epilepsy: implications in hippocampal sclerosis development and drug resistance

**DOI:** 10.3389/fnmol.2024.1512860

**Published:** 2024-12-18

**Authors:** Bárbara Guerra Leal, Cláudia Carvalho, Cristina Santos, Raquel Samões, Ricardo Martins-Ferreira, Catarina Teixeira, Diana Rodrigues, Joel Freitas, Carolina Lemos, Rui Chorão, João Ramalheira, João Lopes, António Martins da Silva, Paulo Pinho e Costa, João Chaves

**Affiliations:** ^1^UMIB-Unit for Multidisciplinary Research in Biomedicine, ICBAS- Instituto de Ciências Biomédicas Abel Salazar da Universidade do Porto, Porto, Portugal; ^2^ITR–Laboratory for Integrative and Translational Research in Population Health, Porto, Portugal; ^3^Immunogenetics Laboratory, Department of Molecular Pathology and Immunology, Instituto de Ciências Biomédicas Abel Salazar da Universidade do Porto (ICBAS-UPorto), Porto, Portugal; ^4^Neurology Department, Hospital de Santo António–Unidade Local de Saúde de Santo António (HSA-ULSSA), Porto, Portugal; ^5^Neurophysiology Department, Hospital de Santo António–Unidade Local de Saúde de Santo António (HSA-ULSSA), Porto, Portugal; ^6^Instituto Nacional Saúde Ricardo Jorge Delegação Norte, Porto, Portugal

**Keywords:** MTLE, microRNAs, biomarkers, DRE, miR-134, GGE

## Abstract

**Aim:**

miR-134 has been widely reported as upregulated in experimental and human studies of Mesial Temporal Lobe Epilepsy the most common drug-resistant epilepsy (DRE). Studies have shown that the use of antagomirs, anti-miR-134, may be a promising therapeutic approach to these epilepsies. However, data on miR-134 in other epileptic syndromes is scarce. In this study, we aimed to quantify serum levels of miR-134 in a cohort of patients with Mesial Temporal Lobe Epilepsy-Hippocampal Sclerosis (MTLE-HS) and with Genetic Generalized Epilepsies (GGE). Additionally, we explored the correlation between miR-134 serum levels and clinical parameters, such as age at onset or febrile seizures antecedents, to evaluate its potential as a biomarker and therapeutic target in epilepsy.

**Methods:**

miR-134 levels were evaluated in cell-free serum of 131 patients with epilepsy (75 women, 56 men; age 41.10 ± 13.12 years; 72 with DRE) and 42 healthy individuals (25 women, 17 men; age 42.40 ± 9.80 years). The epilepsy cohort included 77 MTLE-HS patients and 54 GGE patients.

**Results:**

Patients with elevated miR-134 circulating levels were at higher risk of drug-resistant epilepsy (OR [95% CI] = 2.246 [1.111–4.539], *p* = 0.021). Other risk factors included an older age (OR [95% CI] = 1.032 [1.004–1.061], *p* = 0.025), history of febrile seizures (OR [95% CI] = 2.994 [1.385–6.471], *p* = 0.005) and higher disease duration (OR [95% CI] = 1.038 [1.011–1.066], *p* = 0.006). The strongest predictor of DRE was hippocampal sclerosis (OR [95% CI] = 10.338 [4.566–23.404], *p* < 0.001). Circulating miR-134 levels were significantly higher in MTLE-HS patients compared to controls (*p* < 0.05) and GGE patients (*p* < 0.05). However, the clinical utility of miR-134 in discriminating MTLE-HS patients from controls was only moderated (AUC = 0.651 ± 0.051 95% CI 0.551–0.751, *p* = 0.007).

**Conclusion:**

We show that miR-134 circulating levels are associated with DRE, especially in MTLE-HS, a syndrome characterized by severe hippocampal damage, consistent with activity-regulated miR-134 expression. This overexpression likely contributes to disease progression and our results support the potential of targeting miR-134 as a novel therapeutic approach for refractory epilepsy.

## 1 Introduction

Epilepsy is a chronic neurological disease that affects more than 50 million persons worldwide, 80% of whom live in low-and middle-income countries (World Health Organization, 2019). This is a heterogeneous condition that has no cure. Pharmacological treatments only manage seizure activity but do not address the underlying epileptogenic mechanisms (Perucca et al., 2023). While most patients achieve seizure control with anti-seizure medication (ASMs), more than 30% remain refractory, experiencing recurrent seizures despite treatment (Brodie et al., 2012; Perucca et al., 2023). Refractory seizures are associated with high mortality and morbidity rates (GBD, 2019) imposing substantial social and healthcare costs (Begley et al., 2022). A biomarker for the early and accurate diagnosis of this condition remains yet to be identified. In this context, microRNAs (miRNAs) have emerged as promising candidates (Brindley et al., 2019). These small non-coding RNA molecules regulate gene expression, playing an important role in the modulation of neurotransmission and neuroinflammation (Alsharafi et al., 2015). Different miRNA expression profiles have been observed in epilepsy with over 100 miRNAs found dysregulated (Mooney et al., 2016). Among these, miR-134, a brain-enriched miRNA, has gained special attention (Morris et al., 2019). miR-134 is expressed in neurons and has a key role in the control of neuronal microstructure modulating dendritic spine morphology and consequently synaptic plasticity and development as well as neuronal differentiation and survival (Schratt et al., 2006). miR-134 upregulation has been described in brain and body fluids of experimental and human epilepsy (Jimenez-Mateos et al., 2012; Peng et al., 2013; Wang et al., 2014; Jimenez-Mateos et al., 2015; Reschke et al., 2017; Leontariti et al., 2020). Notably, miR-134 silencing attenuates epilepsy development and progression. The use of antagomirs anti-miR-134, suppresses the occurrence of both evoked and spontaneous seizures (Jimenez-Mateos et al., 2012), reduces seizure-induced neuronal damage (Jimenez-Mateos et al., 2012; Sun et al., 2017; Gao et al., 2019), and decreases the number and volume of dendritic spines, as well as aberrant mossy fiber sprouting (Gao et al., 2019). Most of these studies focus on Mesial Temporal Lobe Epilepsy with Hippocampal Sclerosis (MTLE-HS), the most common form of refractory epilepsy in adults. Over 80% of MTLE-HS cases exhibit poor response to first-line ASMs, highlighting the critical need for alternative therapeutic approaches (Engel, 1998).

Although miR-134 has been proposed as a potential biomarker and therapeutic target for refractory MTLE-HS, it remains unclear whether this dysregulation extends to other epileptic syndromes. This study aims to explore the role of miR-134 role in a broader spectrum of epilepsies. Specifically, we propose to evaluate serum levels of miR-134 in a cohort of refractory patients with MTLE-HS and with Genetic Generalized Epilepsies (GGE). Additionally, we will correlate miR-134 serum levels with clinical variables, such as age at onset or febrile seizures antecedents, to further characterize its potential as a biomarker and / or therapeutic target in epilepsy.

## 2 Materials and methods

### 2.1 Population

**Figure 1:**
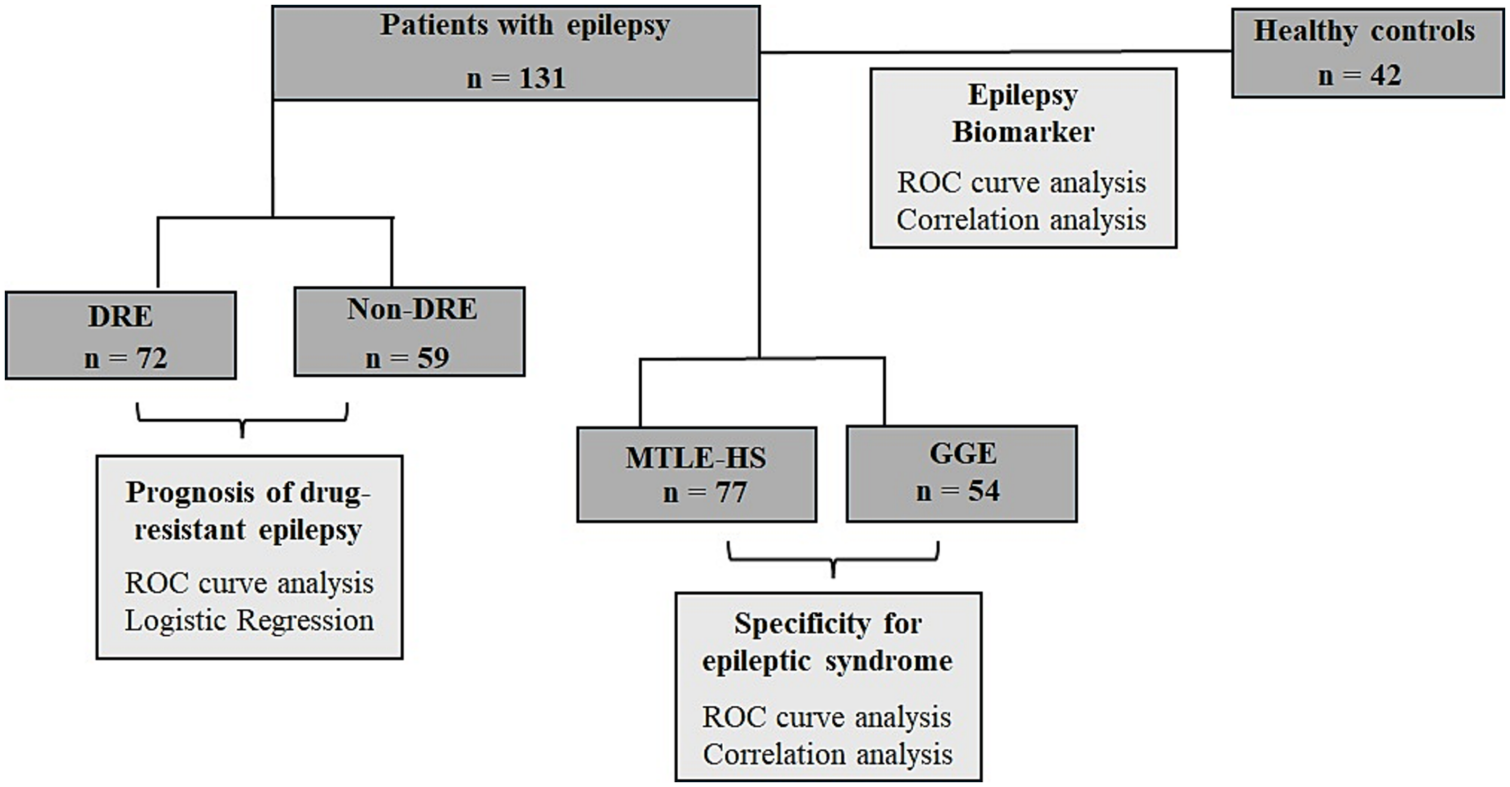
Flow chart of the study design of circulating miR-134 levels in epilepsy patients. Serum levels of miR-134 was quantified in a total of 131 patients with epilepsy and 42 healthy controls were studied. The first approach was to analyze the usefulness of miR-134 as epilepsy biomarker. Next, we want to analyze the usefulness of serum miR-134 levels as predictor of response to ASDs and finally we wanted to analyze if miR-134 upregulation was specific of MTLE-HS.

This study included 131 patients with epilepsy (75 women, 56 men; age 41.10 ± 13.12, Table 1 and Figure 1) and 42 healthy individuals (25 women, 17 men; age 42.40 ± 9.80, Table 1). Patients were recruited from the Neurology Outpatient Clinic of *Hospital Santo António – Unidade Local de Saúde de Santo António* (HSA- ULSSA) a Portuguese Referral Centre for drug-resistant epilepsy, 77 patients with MTLE-HS and 54 patients with GGE Demographic and clinical data were retrieved from medical records. MTLE-HS and GGE diagnosis was based on clinical and electrophysiological studies (EEG and/or video-EEG monitoring) and on brain MRI (minimum 1.5 T) features. In MTLE patients’ diagnosis, hippocampal sclerosis, present in all patients, was based on brain MRI findings which comprised atrophy, T2 hyperintensity signal and altered internal structure in one or both hippocampi associated or not with other imaging criteria, like ipsilateral fornix atrophy, ipsilateral mamillary bodies atrophy or ipsilateral entorhinal abnormalities. Patients with other MTLE-HS aetiologies like HS due to dual pathology were excluded from the study. Computed tomography and/or magnetic resonance brain imaging were normal in all GGE patients.

**Table 1:** Clinical and demographic features of the subgroups of patients with epilepsy considered in the analysis.

Variable	DRE	Non-DRE	MTLE-HS	GGE
	(*n* = 72)	(*n* = 59)	(*n* = 77)	(*n* = 54)
Gender, *n* (%)				
Male	34 (47.22)	22 (37.29)	33 (42.86)	23 (42.59)
Female	38 (52.78)	37 (62.71)	44 (57.14)	31 (57.419)
Febrile Seizures antecedents, *n* (%)				
No	39 (54.17)	46 (77.97)	38 (49.35)	47 (79.66)
Yes	33 (45.83)	13 (22.03)	39 (50.65)	7 (11.86)
Hippocampal sclerosis, *n* (%)				
No	13 (18.06)	31 (52.54)	0 (0.00)	54 (100.00)
Yes	59 (81.94)	18 (30.51)	77 (100.00)	0 (0.00)
ASM, *n* (0/mono/polytherapy)	1/12/59	5/33/21	1/14/62	5/31/18
Age, mean ± SD (range), years	43.49 ± 12.62 (21–78)	38.25 ± 13.26 (13–74)	44.22 ± 12.85 (13–78)	44.20 ± 12.85 (18–64)
Age at onset, mean ± SD (range), years	13.57 ± 7.43 (0–32)	15.19 ± 12.10 (1–55)	15.14 ± 11.14 (0–55)	13.09 ± 6.89 (1–37)
Disease Duration, mean ± SD (range), years	29.92 ± 14.06 (3–58)	23.07 ± 12.93 (2–56)	29.08 ± 14.23 (2–58)	23.63 ± 12.99 (2–54)

SD, standard deviation. Polytherapy considered when number ASM is equal or higher than 2 in a maximum of 4.

Following International League against Epilepsy (ILAE) guidelines, DRE was defined as the occurrence of frequent seizures despite treatment with at least two appropriated ASMs at adequate doses either in mono or polytherapy (Kwan et al., 2010). At the time of the study all but six patients (1 patient with MTLE-HS and 5 with GGE) were under ASM and 72 patients were classified as having Drug-Resistant Epilepsy (Table 1). For MTLE-HS the most used medication in mono or polytherapy was carbamazepine (45.8% MTLE-HS vs. 6% GGE) whilst for GGE was valproate (17% MTLE-Hs vs. 61.2% GGE).

Control individuals were voluntarily recruited among blood donors, ethnically matched, from the same geographic region. This study was approved by the Ethics Committee of the participating institutions [CE CHUP/ICBAS] and conducted in accordance with the local legislation and institutional requirements. All the participants provided their written informed consent to participate in this study complying with the World Medical Association Declaration of Helsinki.

### 2.2 Blood collection

Peripheral blood samples were collected in Vacuette Serum Sep Clot Activator tubes, centrifuged at 1500 g for 15 min at room temperature and serum aliquots were stored at −20°C. Samples processed more than 4 h post-collection were excluded. Haemolysis was visually evaluated based on the sample color. Any sample with signs of haemolysis (with an orange to red tinge) was assessed by spectrophotometric analysis using Nanodrop 2000 spectrophotometer. Samples with an absorbance at 414 nm higher or equal at 0.2 were not included in the study due to the possibility of haemolysis. RNA was extracted using the miRNeasy® Serum/Plasma Kit (Qiagen, Germany), according to the manufacturer’s protocol.

### 2.3 miR-134 quantification by real time–PCR

Five ng of RNA were converted to cDNA with the Taqman® miR Reverse Transcription Kit (4,366,597 - Applied Biosystems, USA) and Taqman® miR Assays (001186, Applied Biosystems, USA). The reaction was performed in a Biometra Alfagene® thermocycler according to the manufactures’ instructions. The quantitative RT-PCR amplification was run with a NzySpeedy qPCR mastermix (Nzytech, Portugal) in a Corbett Rotor Gene 600 Real Time Thermocycler (Corbett Research, UK). One μL of cDNA was used per reaction and triplicates were run for each sample. miR-134 levels were evaluated in serum, a cell-free body fluid with no known RNA species at constant levels that could be used as housekeeping genes for normalization (Wang et al., 2010). Briefly, to overcome this issue, reactional conditions and serum sample volumes were constant and uniform throughout all assays, and the same baseline and threshold cycle was set, so that expression levels could be comparable between samples. Circulating miR-134 levels are expressed as individual 50-Ct arbitrary units (Wang et al., 2010; Martins-Ferreira et al., 2020).

### 2.4 Statistical analysis

A flowchart detailing the study rationale, including the groups considered and the analyses performed, is presented in Figure 1.

Relative expression was calculated based on the 2^–ΔΔCT^ method. Differences in ΔCt (50 – Ct) were evaluated using a two-tailed Student’s t-test or Mann–Whitney test as appropriated. To account for multiple testing, ANOVA or Kruskal-Wallis tests followed by Tukey’s and Dunn’s corrections, respectively, were used. Temporal variations within the same group were analyzed with paired-samples Student’s T-test or Wilcoxon test as appropriated. Correlations between miR-134 circulating levels and continuous variables including age, age at onset and disease duration were assessed using Spearman’s test. The diagnostic performance of miR-134 circulating levels was evaluated through logistic regression analysis and Receiver Operating Characteristic (ROC) curves, which plot the sensitivity (true positivity rate) against the 1-specificity (false positivity rate). Diagnostic accuracy was quantified by calculating the area under the curve (AUC). To assess the independent clinical significance of circulating miR-134 levels multivariate logistic regression models were adjusted for both clinical (febrile seizure antecedents, hippocampal sclerosis, age at onset and disease duration) and demographic (age and gender) variables. Odds ratios (ORs) and their corresponding 95% confidence intervals (CIs) were calculated to estimate the strength of the associations. Internal validation of the regression models was performed by bootstrap logistic regression analysis based on 1,000 bootstrap samples. The 45th percentile was used as the optimal cut-off value of miR-134 levels.

Statistical analysis was performed with the SPSS (Statistics Package for Social Sciences) software version 29.0. Graphs were developed with GraphPad Prism 9.01. Significant levels were set at *p* < 0.05 for all statistical analyses.

## 3 Results

**Figure 2:**
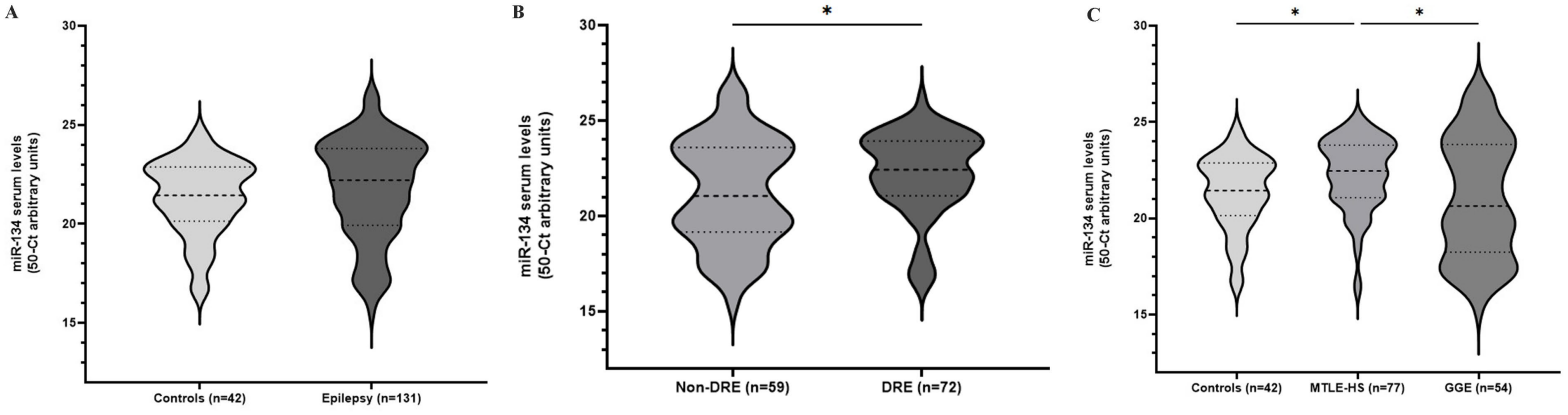
Circulating miR-134 levels in epileptic patients and controls. Violin plots represent pooled data from 42 blood donor controls and 131 patients with epilepsy **(A)**; Patients were subdivided according to drug-response **(B)** or epileptic syndrome **(C)**. Mann–Whitney **(A,B)** and Kruskal-Walls with Dunn’s correction for multiple testing **(C)** were applied. *p < 0.05 represent significant differences.

**Figure 3:**
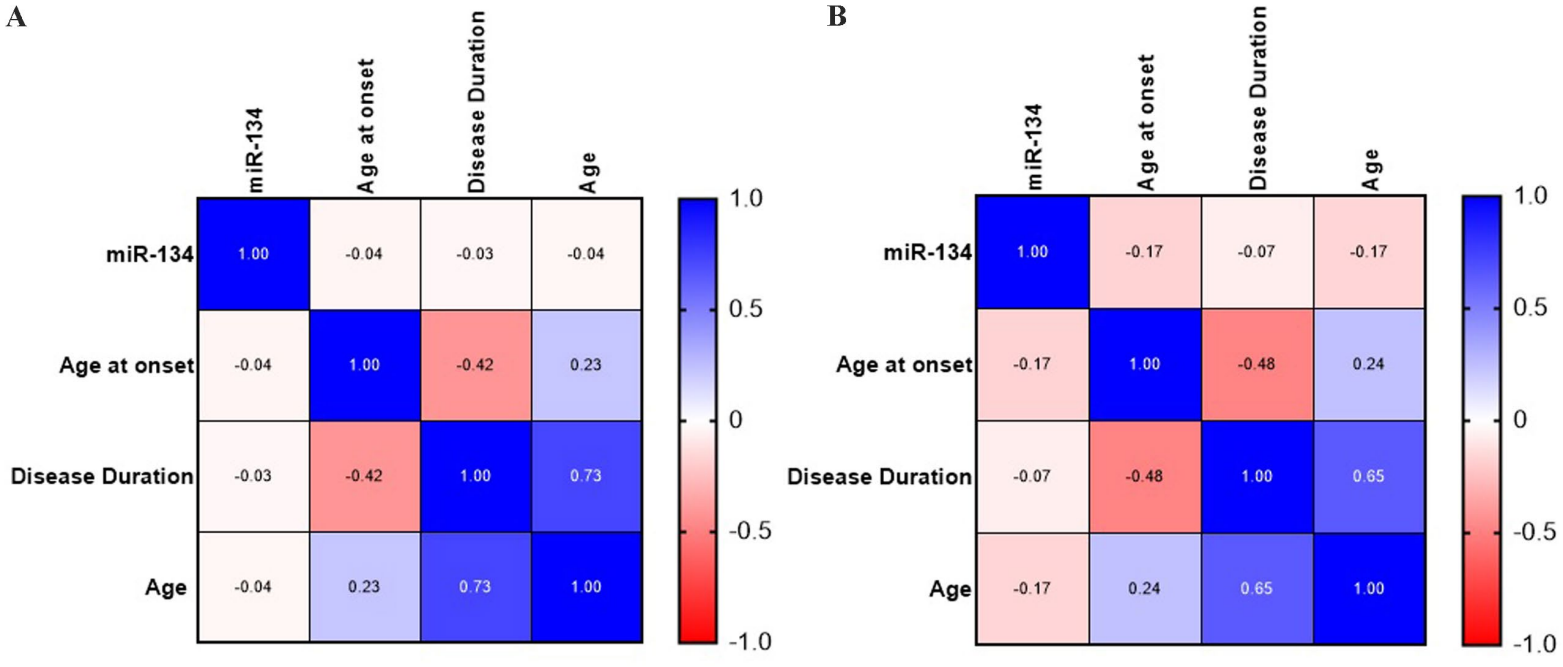
Correlation analysis of circulating miR-134 levels and clinical and demographic variables for general epilepsy **(A)** and MTE-HS **(B)**. No significant correlation was observed between circulating miR-134 levels and clinical and demographic variables as age, age at onset and disease duration both in general epilepsy and MTLE-HS patients. Continuous Variables were used. Heatmaps of Spearman’s correlation coefficients were created with GraphPad Prism.

**Figure 4:**
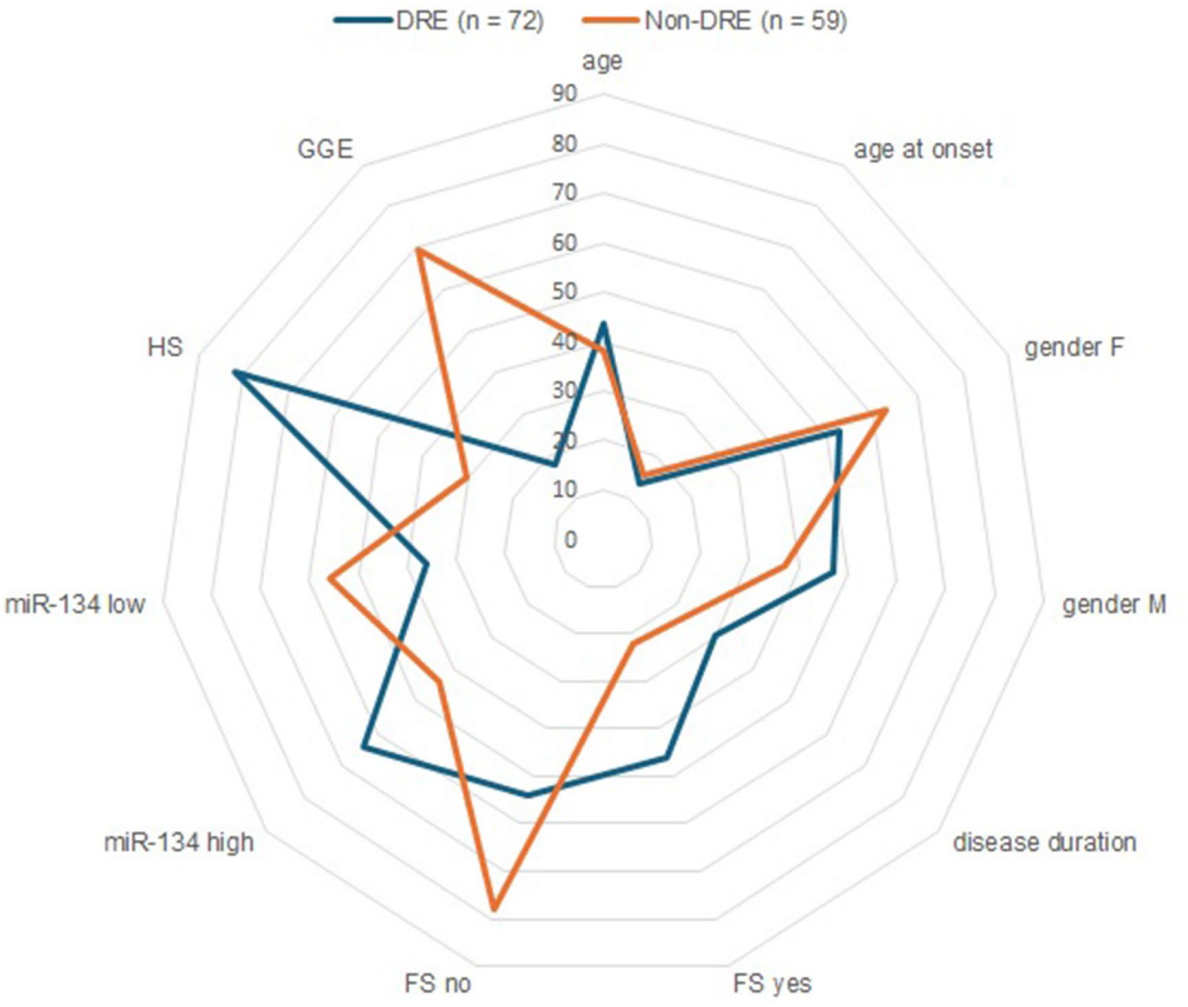
Circulating miR-134 clinical and demographic variables as predictors of refractrory epilepsy. Radar plots compares refractory (blue line) and non-refractory (orange line) epilepsy patients in relation to dichotomous (Gender, Febrile Seizures, Hippocampal Sclerosis, serum miR-134 levels) and continuous (age, age at onset, disease duration, presented as mean). The 45th percentile was used as optimal cut-off values of miR-134 levels.

miR-134 circulating levels did not significantly differ between the control group and the general cohort of epilepsy patients (fold-change: 1.36 *p* > 0.05, Figure 2A). Receiver operating characteristic curve analysis showed a low AUC of 0.575 ± 0.045 95% CI 0.487–0.663, p > 0.05, Table 2) indicating a limited value for miR-134 in discriminating patients with epilepsy from healthy individuals. No correlations between miR-134 serum levels and continuous clinical variables (age at onset, disease duration and age) were observed for epilepsy patients (Figure 3A). Two subgroups of patients were analyzed based on their response to ASMs: 72 patients with DRE and 59 patients who responded well to pharmacological treatment (non-DRE). Patients with DRE had higher miR-134 circulating levels than non-refractory patients (*p* = 0.027, Figure 2B). The diagnostic value of miR-134 in predicting DRE was moderate as indicated by the ROC curve analysis (AUC = 0.613 ± 0.051, 95% CI 0.513–0.713, *p* = 0.027; Table 2). Nevertheless, the univariate logistic regression showed that patients with higher circulating miR-134 levels were at higher risk of drug-refractoriness (OR [95% CI] = 2.246 [1.111–4.539], *p* = 0.021, Table 3 and Figure 4). Additionally, it was also showed that older patients (OR [95% CI] = 1.032 [1.004–1.061], *p* = 0.025), those with a history of FS antecedents (OR [95% CI] = 2.994 [1.385–6.471], *p* = 0.005) and those with a higher disease duration (OR [95% CI] = 1.038 [1.011–1.066], *p* = 0.006) were also at higher risk of developing DRE (Table 3 and Figure 4). Hippocampal Sclerosis was the strongest predictor of DRE ((OR [95% CI] = 10.338 [4.566–23.404], *p* < 0.001, Table 3 and Figure 4). This was the only result that remained significant in the multivariate analyses (OR [95% CI] = 10.338 [4.566–23.404], *p* < 0.001, Table 4).

**Table 2:** Receiver operator characteristic for diagnostic ability of circulating miR-134 levels for the different considered outcomes.

ROC	Epilepsy *vs*	DRE *vs*	MTLE *vs*	MTLE *vs*	GGE *vs*
	controls	Non-DRE	Controls	GGE	Controls
AUC	0.575	0.613	0.651	0.624	0.534
SE	0.045	0.051	0.051	0.055	0.060
95% CI	0.487–0.663	0.512–0.713	0.551–0.751	0.516–0.731	0.415–0.652
*p*-value	0.15	0.027	0.006	0.58	0.57

AUC, area under the curve; SE, standard error; 95% CI, 95% confidence interval.

**Table 3:** Univariate logistic regression analysis for the prediction of Drug-resistant Epilepsy according to circulating miR-134 levels.

Co-variant	OR	95% CI	*p*-value	Bootstrap Bca 95% CI	Bootstrap *p*-value
Gender					
Male	1.000				
Female	0.665	0.329–1.341	0.254	−1.103 – 0.336	0.246
Febrile seizures					
No	1.000				
Yes	2.994	1.385–6.471	**0.005**	0.306–2.082	0.007
Hippocampal sclerosis					
No	1.000				
Yes	10.338	4.566–23.404	**<0.001**	1.541–3.375	<0.001
Disease duration	1.038	1.011–1.066	**0.006**	0.011–0.71	0.008
Age at onset	0.983	0.949–1.019	0.348	−0.051 - 0.028	0.346
Age	1.032	1.004–1.061	**0.025**	0.004–0.065	0.025
miR-134 serum					
Low levels	1.000				
High levels	2.246	1.111–4.539	**0.021**	0.061–1.612	0.021

OR, odds ratio; 95% CI, 95% Confidence Interval; Bootstrap Ca, Bootstrap bias-corrected and accelerated 95% confidence interval of the OR based on 1,000 bootstrap samples. Statistical significant differences (*p*-value <0.05) are indicated in bold.

**Table 4:** Multivariate logistic regression analysis for the prediction of Drug-resistant Epilepsy.

Co-variant	OR	95% CI	*p*-value	Bootstrap Bca 95% CI	Bootstrap *p*-value
Febrile seizures					
No	1.000				
Yes	1.181	0.444–3.146	0.739	−0.949 – 1.256	0.733
Hippocampal sclerosis					
No	1.000				
Yes	8.987	3.434–23.517	**<0.001**	1.092–3.743	<0.001
Disease Duration	1.044	0.998–1.091	0.059	−0.017 – 0.091	0.056
Age	0.982	0.935–1.032	0.475	−0.072 – 0.054	0.497
miR-134 serum					
Low levels	1.000				
High levels	1.709	0.738–3.956	0.211	−0.555 – 1.75	0.198

Multivariate Logistic regression was adjusted for FS antecedents, Age, Disease Duration, Temporal Sclerosis and miR-134 levels which were significant in the univariate logistic regression. OR, odds ratio; 95% CI, 95% Confidence Interval; Bootstrap Ca, Bootstrap bias-corrected and accelerated 95% confidence interval of the OR based on 1,000 bootstrap samples. Statistical significant differences (*p*-value <0.05) are indicated in bold.

Based on these findings we analyzed MTLE-HS and GGE cohorts independently to assess differential miR-134 expression. Circulating miR-134 levels were significantly higher in MTLE-HS patients comparing to controls (*p* < 0.05, Figure 2C) and to GGE patients (*p* < 0.05, Figure 2C). However, miR-134 had only a moderate clinical value in discriminating MTLE-HS patients from controls (AUC = 0.651 ± 0.051 95% CI 0.551–0.751, *p* = 0.007, Figure 3B and Table 2). No correlations between miR-134 serum levels and clinical variables as age at onset, disease duration and age were observed for MTLE-HS patients (Figure 3B). No significant differences were observed between patients with GGE and controls (*p* > 0.05, Figure 2C).

## 4 Discussion

The biological mechanisms underlying drug-resistant epilepsy development are yet to be identified, although seizure activity has been proposed as a key contributing factor (for review see Perucca et al., 2023; Remy and Beck, 2006). In this study, we found that longer epilepsy duration is a significant predictor of the development of refractory epilepsy, supporting the hypothesis that sustained seizure activity drives disease progression. Recurrent and severe seizures lead to structural damage which triggers additional seizures, in a self-perpetuating cycle of neuronal damage and excitotoxicity. This not only exacerbates epilepsy but also can also predispose to the development of several comorbidities, which are in turn associated with a worse prognosis (Giussani et al., 2021). Frequent seizures have been associated with cognitive decline, mood fluctuations, and personality issues, as well as with increased risk of sudden death. Moreover, seizure activity can induce changes in the molecular targets of ASMs reducing their efficacy. A classic example is the development of hippocampal sclerosis, which is characterized, among other features, by the loss of neurons in the hippocampus. MTLE-HS is the most common refractory epilepsy syndrome in adults and, as demonstrated in our study, the strongest predictor of poor response to ASMs. These findings underscore the importance of early intervention to control seizure activity highlighting the importance of prompt identification of at-risk patients.

For refractory cases, alternative non-pharmacological interventions including dietary strategies such as ketogenic diet or neurostimulation techniques may be considered albeit with moderate efficacy. Surgical resection of the seizure foci is the most common intervention for focal epilepsies. However, it involves a costly and time consuming pre-surgical evaluation and has limited efficacy with over 35% of patients reporting seizure recurrence several years after surgery (Jeha et al., 2007; Hemb et al., 2013). Thus, the effective resolution of seizures remains an unmet clinical need.

In the last decade, microRNAs have emerged as attractive research subjects in epilepsy. These are pleiotropic molecules that modulate gene expression (Ambros, 2004), having a critical role in fine-tunning neurotransmission, synaptic plasticity and neuroinflammation, all of which are fundamental in epileptogenesis (Henshall et al., 2016; Brennan and Henshall, 2020). In both experimental and human epilepsy over 100 microRNAs have been described as dysregulated (Mooney et al., 2016) supporting the hypothesis that these molecules can give important insights into the molecular mechanisms underlying seizure development and progression. MicroRNAs are highly stable in biological fluids where they are thought to reflect tissue production (Brindley et al., 2023). Moreover, their quantification in biological fluids can be performed through time- and cost-efficient methods, making miRNAs attractive candidates as biomarkers of epilepsy diagnosis, progression and therapeutic response (Brindley et al., 2019). In this context miR-134 is one of the most extensively studied miRNAs, predominantly found upregulated in brain and circulating fluids. Our results are consistent with the literature as we described higher miR-134 serum levels in DRE patients, particularly in those with MTLE-HS (Jimenez-Mateos et al., 2012; Peng et al., 2013; Leontariti et al., 2020). Nevertheless, the diagnostic performance, as assessed by ROC curve and AUC analysis, was only moderated. These results suggest that while miR-134 may not be a reliable biomarker for MTLE-HS or the development of DRE it can provide valuable insights into dysregulated epileptogenic pathways or even poses as a new therapeutic target. miR-134 is a brain-enriched miRNA, highly expressed in dendrites that plays an important role in the modulation of neuronal microstructure (Schratt et al., 2006). Its upregulation in cases of recurrent seizures, as observed in DRE in our study, may be a response to increased neuronal activity (Wang et al., 2014; Gao et al., 2019), as miR-134 modulates activity-dependent dendrite growth, development and plasticity (Schratt et al., 2006). Consistent with our findings, Jimenez Mateo et al. reported that miR-134 overexpression was detected only in chronic epilepsy and damage-inducing seizures, whilst non-harmful seizures were not sufficient to alter miR-134 expression (Jimenez-Mateos et al., 2012). Similarly, Peng et al., using a an animal model of epilepsy, demonstrated that miR-134 expression is upregulated in the acute and chronic phase of epileptogenesis but remains similar to control levels during the latent phase (Peng et al., 2013). In agreement with these findings, we observed that miR-134 upregulation seems to be specific of MTLE-HS, a syndrome associated with severe hippocampal damage (Blumcke et al., 1999). miR-134 upregulation was not detected in our GGE cohort suggesting that patients with GGE may be more protected against damage-inducing seizures avoiding dendritic injury and dysregulated pathways associated with miR-134 upregulation. On the other hand, anti-seizure medications are known to influence epigenetic mechanisms including microRNA expression (Navarrete-Modesto et al., 2019). Wang et al. demonstrated that miR-134 plasma levels were downregulated after 1 month of valproate treatment (Wang et al., 2017), the most used ASM in GGE patients within our cohort. This may account for the lower miR-134 levels in GGE patients compared to MTLE-HS patients observed in our study. Future studies should include a validation cohort that includes other epilepsy syndromes and diverse ASM regimens to determine whether miR-134 upregulation is specific to MTLE-HS or represents a common feature across various epilepsy syndromes.

Studies in animal models demonstrated that miR-134 overexpression reduces spine volume (Schratt et al., 2006), decreases total dendritic length (Christensen et al., 2010) and impairs long-term potentiation, thus negatively affecting synaptic plasticity (Gao et al., 2019). All of this contribute to the development of the cellular and molecular abnormalities that characterize hippocampal sclerosis. These effects are mediated through the inhibition of LIM kinase 1 (Limk1) and Pumilio-2 (Pum2), known molecular targets of miR-134. Limk1 is a serine/threonine kinase highly expressed in hippocampal pyramidal neurons where it plays a modulation role in actin dynamics regulating dendritic spines morphology and development. Knockout models of Limk1 demonstrate abnormal dendritic morphology and synaptic dysfunction (Meng et al., 2003). Pum2 is an RNA-binding protein, predominantly localized in neuron dendrites (Vessey et al., 2006), that is essential for the modulation of synaptic plasticity (Vessey et al., 2010). Loss of Pum2 has been shown to promote dendritic outgrowth and arborization and increase excitatory synapses (Vessey et al., 2010). Noteworthy, Limk1 and Pum2 expressions have been found to follow an opposite pattern of miR-134 in both experimental and human cases (Jimenez-Mateos et al., 2012; Wu et al., 2015; Follwaczny et al., 2017). In addition to Limk1 and Pum2, other molecular targets of miR-134 include CREB which is involved in synaptic plasticity and neuronal death (Gao et al., 2019).

Our findings of elevated miR-134 levels in patients with hippocampal sclerosis, combined with evidence from studies in animal models suggest the existence of vicious cycle between damage-inducing seizures and miR-134 overexpression. Increased neuronal activity leads to cellular damage which in severe cases culminates in hippocampal sclerosis. As part of the damage repair mechanism and in response to neuronal activity, miR-134 expression is upregulated to modulate dendritic spines development and neuronal microstructure. However, in susceptible individuals, dysregulation of these processes may occur with miR-134 overexpression disrupting synaptic transmission and dendritic morphology. This dysfunction exacerbates neuronal excitability promoting seizure recurrence and further neuronal damage which in turn enhances miR-134 expression. Over time, this self-perpetuating cycle may drive the progression of HS and contribute to the development of DRE, emphasizing the role of miR-134 in epilepsy progression and treatment response.

In line with this it has been observed that miR-134 silencing using antagomirs, has beneficial effects in different *in-vivo* and *in-vitro* models (Jimenez-Mateos et al., 2015; Sun et al., 2017; Gao et al., 2019; Morris et al., 2019; Campbell et al., 2021; Campbell et al., 2022). Ant-134 injection prior to status epilepticus (SE) induction reduces the severity and recurrence of seizures (Jimenez-Mateos et al., 2012; Wang et al., 2014; Reschke et al., 2017; Gao et al., 2019), and extends the latent period with more seizure-free days (Jimenez-Mateos et al., 2015; Reschke et al., 2017; Morris et al., 2019), The protective effects of anti-miR-134 treatment are likely due to histological improvements including reduced seizure-induced damage and decreased number of apoptotic cells (Gao et al., 2019). In fact, treatment with anti-miR-134 has been shown to preserve hippocampal morphology by maintaining normal neuronal counts, reducing astrogliosis (Jimenez-Mateos et al., 2012; Sun et al., 2017) and preventing mossy fiber sprouting (Gao et al., 2019). Hippocampal dendritic spine density was also reduced (Jimenez-Mateos et al., 2012) and autophagy and lipid peroxidation markers were normalized (Sun et al., 2017) These findings were accompanied by the restoration of Limk1 and Creb1 expressions to normal levels (Jimenez-Mateos et al., 2012).

Noteworthy, Jiménez-Mateo et al. showed that while ant-134 injection following SE induction did not affect the acute phase of SE, it exerts a neuroprotective effect leading to a significant reduction in seizure frequency several weeks post-injection (Jimenez-Mateos et al., 2012). A similar finding was reported for another epilepsy model, where epilepsy was prevented in over 85% of animals treated with ant-134 (Reschke et al., 2017). These observations suggest an anti-epileptogenic effect of silencing miR-134, as the sustained seizure reduction is maintained even in the absence of ongoing antagomir administration (Jimenez-Mateos et al., 2012). Although it has been demonstrated that ant-134 specifically inhibits miR-134 without silencing other miRNAs (Morris et al., 2022) and that its administration does not impact normal brain anatomy and cognitive behavior (Morris et al., 2018) several concerns remain regarding the use of miR-134 silencing as a therapeutic approach for human epilepsy. It has been argued that miR-134 role in modulating dendritic spines and synaptic plasticity may not only be regulated by neuronal activity but could also be coupled with pathological brain damage (Jimenez-Mateos et al., 2012). This regulation may be modulated by the cellular and molecular environment (Gao et al., 2019) including other molecules and miRNAs. For instance, Leontariti et al. demonstrated that the combination of elevated miR-134 and miR-146a levels was associated with a higher risk of developing DRE than either miRNA alone (Leontariti et al., 2020), suggesting that both neuronal microstructure dysfunction and neuroinflammation contribute to DRE development. Given that miR-134 silencing promotes seizure reduction, it is plausible that it indirectly mitigates neuroinflammation potentially influencing miR-146a levels. This indirect effect of miR-134 silencing on other miRNAs has also been described by Raoof et al. (2018). These observations suggest that the disease-modifying effects of miR-134 silencing may be sustained through epigenetic changes, further highlighting its potential in epilepsy management.

Despite that miR-134 upregulation is considered “a conserved molecular response to seizures” (Jimenez-Mateos et al., 2015) not all studies report consistent observations. Some miRNA profiling studies in blood or brain tissues of animal models have reported no significant alterations in miR-134 levels (Liu et al., 2010; Hu et al., 2011; Risbud and Porter, 2013). This may be due to temporal variations in sampling as miRNAs expression can be time dependent. Additionally, Avansini et al. observed the downregulation of miR-134 plasma levels in human Temporal Lobe Epilepsy, contrasting with our finding (Avansini et al., 2017). These discrepancies may be due to differences in the patient cohorts - in our study all patients had Hippocampal Sclerosis - or to the type of biological samples analyzed.

A limitation of our study is the lack of validation cohorts. The inclusion of a larger independent cohort of MTLE-HS patients would strength our findings as would the study of other epilepsy syndromes. Additionally, it would be interesting to study the effects of ASMs on circulating miR-134 levels particularly considering that valproate has been reported to downregulate plasma levels of miR-134. This analysis could be conducted in a group of non-epileptic individuals who are taking ASMs for other medical reasons.

In conclusion, our study reveals a significant upregulation of miR-134 in MTLE-HS patients, particularly in those who are refractory to pharmacological treatment. This upregulation, likely triggered by damage-inducing seizures, may downregulate key molecules essential for dendrite structure, function and neuronal survival. Such dysregulation can lead to abnormal hippocampal organization, increased neuronal death and excitotoxic cell damage ultimately promoting recurrent seizures and reducing the responsiveness to ASMs.

Thus, our study highlights the importance miR-134 as a potential epileptogenic intermediate involved in the development of hippocampal sclerosis and in ASM resistance pathways. These findings align with existing literature and underscore the need for preclinical trials aimed at targeting miR-134 with antagomirs to prevent the development of DRE. Furthermore, we propose that clinical variables as disease duration, FS antecedents and HS as well as demographic characteristics like age should be considered in the evaluation of potential refractory cases.

## Data Availability

The raw data supporting the conclusions of this article will be made available by the authors, without undue reservation.
